# Initial ablation ratio predicts the recurrence of low-risk papillary thyroid microcarcinomas treated with microwave ablation: a 5-year, single-institution cohort study

**DOI:** 10.1530/EC-23-0128

**Published:** 2023-08-11

**Authors:** Yujie Ren, Xue Han, Yujiang Li, Guofang Chen, Lin Jiang, Chao Liu, Shuhang Xu

**Affiliations:** 1 Endocrine and Diabetes Center, The Affiliated Hospital of Integrated Traditional Chinese and Western Medicine, Nanjing University of Chinese Medicine, Nanjing, China; 2 Jiangsu Province Academy of Traditional Chinese Medicine, Nanjing, China; 3 Key Laboratory of TCM Syndrome and Treatment of Yingbing (Thyroid Disease) of State Administration of Traditional Chinese Medicine, Jiangsu Province Academy of Traditional Chinese Medicine, Nanjing, China; 4 Department of Endocrinology, The First Affiliated Hospital with Nanjing Medical University, Nanjing, China

**Keywords:** microwave ablation, recurrence, papillary thyroid microcarcinoma, initial ablation ratio

## Abstract

**Objective:**

To assess the long-term efficacy and safety of microwave ablation (MWA) in treating low-risk papillary thyroid microcarcinomas (PTMC) and to identify predictive factors for the postoperative local tumor progression of PTMC.

**Methods:**

A total of 154 low-risk PTMC patients treated with MWA who were followed up for at least 3 months were retrospectively recruited. Ultrasonography was performed after MWA to assess the local tumor progression. Adverse events associated with MWA were recorded. The ablated volume (Va) and initial ablation ratio (IAR) were measured to assess their influences on the recurrence risk of PTMC.

**Results:**

The mean tumor volume of PTMC before MWA was 0.071 (0.039, 0.121) cm^3^, with a maximum diameter of 0.60 ± 0.18 cm. All PTMC patients were followed up for 6 (3, 18) months. Va increased immediately after MWA, then gradually decreased over time, till significantly smaller at 12 months than that before MWA (*P* < 0.05). The median volume reduction ratio at 24 months reached 100%, which was maintained during a 60-month follow-up. A total of 7 (4.55%) cases of local tumor progression were recorded during the follow-up. Kaplan–Meier survival analysis revealed that the rate of local tumor progression was significantly lower in PTMC patients with a maximum tumor diameter < 0.70 cm than in those with ≥0.70 cm (*P* = 0.031). A significant better prognosis was achieved in PTMC patients with IAR ≥ 15 than in those with IAR < 15 (*P* = 0.015). Sex, age (<55 years) and preoperative thyroid-stimulating hormone (>2.0 mU/L) of PTMC patients were not correlated with local tumor progression.

**Conclusion:**

MWA is an effective therapeutic strategy for low-risk PTMC with high safety. The maximum tumor diameter and IAR are predictive factors for the local tumor progression of PTMC after MWA.

## Introduction

With the widespread application of ultrasonography in recent decades (1), the prevalence of thyroid cancer has gradually risen to the 11th and 5th ranks in all cancers and those in women, respectively (2). Papillary thyroid carcinomas (PTCs) are the most common type of thyroid cancer, especially papillary thyroid microcarcinomas (PTMCs) with a diameter ≤ 10 mm. According to ‘Thyroid Cancer Incidence and Mortality Trends in the United States: 2000–2018’, the overall incidence of thyroid cancer in the United States is 11.95/100,000, of which 89.1% and 35.3% are PTCs and thyroid cancers with a diameter ≤ 1 cm, respectively (3). The incidence of thyroid diseases in China has increased significantly as well. A sharp increase in the proportion of PTMC in PTC cases has been observed: from 0 in 1997–1985 to 32.1% in 2000–2014 (4). A retrospective study in Shanghai, China, showed that PTC (98%, 2440/2490) accounts for the majority of thyroid cancers, and more than 50% (1295/2440) of them are diagnosed as PTMCs (5).

Thyroid cancer is characterized by a favorable prognosis and low tumor-specific mortality (6). Low-risk PTMC patients usually have an acceptable prognosis, with 10-year and 15-year survivals of 94.6% and 90.7%, respectively, and the disease-specific survival is up to 99.5% (7). Surgical treatment is preferred for PTMC patients. A recent study has reported that the recurrence rate after unilateral lobectomy in PTMC patients is only 3.8% (8). A meta-analysis of 21,329 person-years of follow-up showed that the postoperative recurrence of nonincidental PTMC is as low as 7.9% (9). Furthermore, the recurrence of nonincidental PTMC is not correlated with age, sex, tumor size, tumor multifocality, lymph node involvement, and therapeutic strategies. Interestingly, a meta-analysis involving 13 clinical studies has demonstrated that male gender, multifocality, tumor size > 5 mm, and extrathyroidal extension are reliable predictors of central lymph node metastasis in CN0 PTMC (clinical node-negative PTMCs) (10). Multiple cohort studies conducted in Japan (11), the United States (12), and Korea (13) have proven that active surveillance (AS) is an alternative to surgical treatment for low-risk PTMC. A Japanese research group has reported that 5 and 10% of PTMC patients experience a minimum increase of 3 mm in the maximum tumor diameter at 5 and 10 years, respectively (14, 15). A multi-center cohort study has shown that tumor volume increase and lymph node metastases are observed in 23.2 and 4.6% of PTMC patients during the AS period. Age is believed to be a prognostic factor for thyroid cancer, that is, younger age is correlated with tumor growth (16). Whether sex is a risk factor for lymph node metastasis in low-risk PTMC, however, is controversial (17).

Thermal ablation is an alternative to low-risk PTMC patients who refuse to be managed by surgery or AS. It may emerge as radiofrequency ablation (RFA), laser ablation (LA), and microwave ablation (MWA), all of which are highly effective and safe for low-risk PTMC. Yan *et al.* (18) have analyzed 884 PTMC patients intervened by surgery or RFA via propensity score matching. They have found that the rate of local tumor progression in PTMC patients treated with RFA is 2.0% (9/460), which is comparable to that in surgically treated patients. However, they did not analyze the relevant risk factors for the local tumor progression of PTMC. Kim *et al.* (19) have followed up on 90 PTMC patients after LA for 10 years. The 12-month volume reduction ratio (VRR) and recurrence rates are 100 and 5.5%, respectively. One patient developed lymph node metastasis shortly after LA. Besides, the recurrence of PTMC is not correlated with age, sex, tumor size, tumor volume, tumor location, presence or absence of adjacent organs, ultrasound features, ablation volume, and ablation time. Wei *et al.* (20) have reported that the disease progression rate of PTMC patients after MWA is 3.4%, and they did not identify any independent risk factors for the progression of T1N0M0 PTC. Our previous study has consistently validated the safety of MWA in the treatment of PTMC, with a 2-year tumor progression rate of 5.48% (4/73) (21). Nevertheless, the risk factors for the recurrence of PTMC after thermal ablation still remain unclear.

The present single-center cohort study analyzed the efficacy and safety of MWA in the treatment of PTMC. Through a 5-year follow-up, we identified that the maximum tumor diameter was a risk factor for the local tumor progression of PTMC, and initial ablation ratio (IAR) was a predictive factor. Our findings support the efficacy of thermal ablation in treating PTMC and preventing postoperative tumor progression.

## Materials and methods

### Subjects

**Figure 1:**
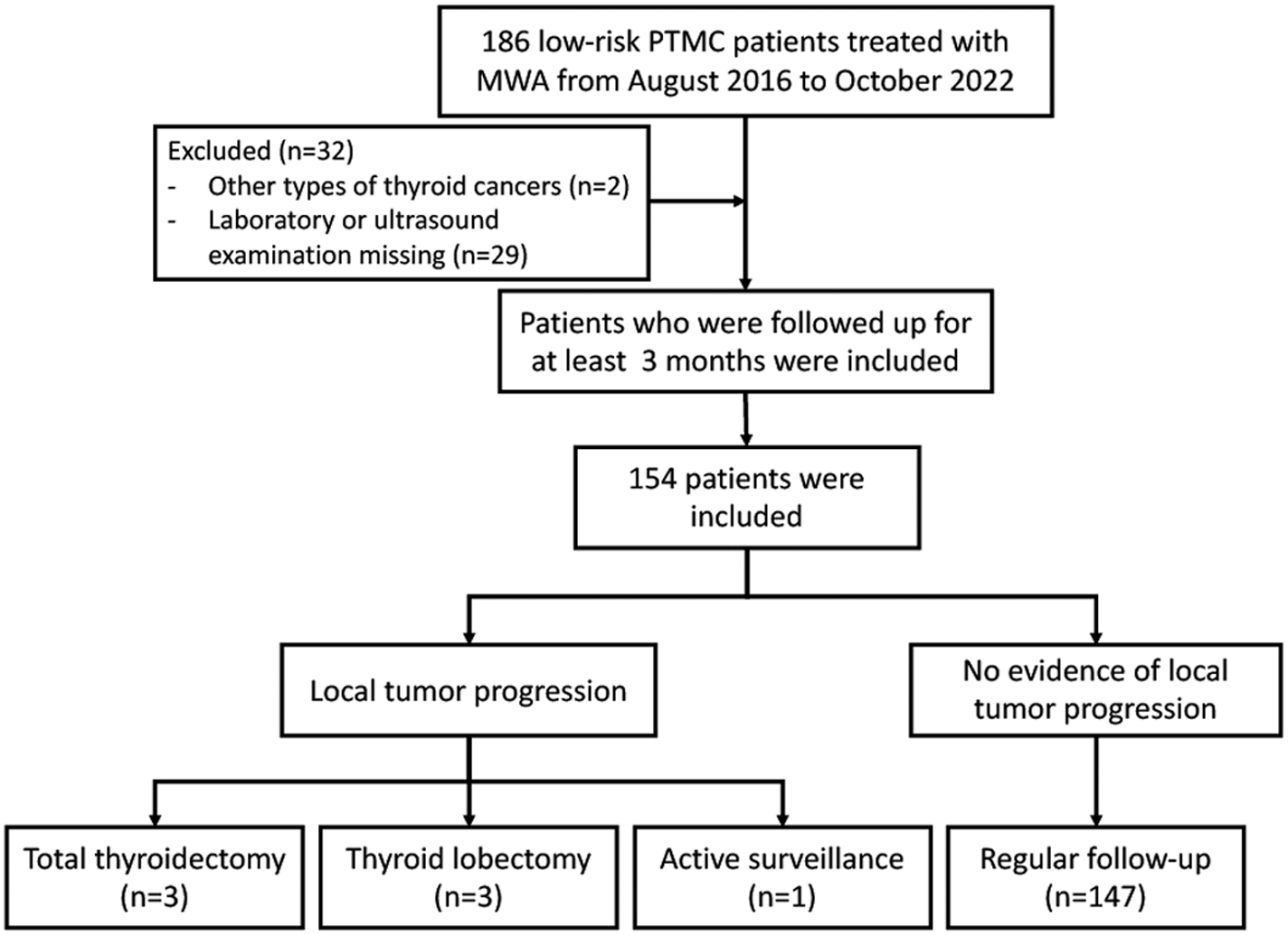
Flow chart of the study cohort. MWA, microwave ablation; PTMC, papillary thyroid microwave ablation.

It was a single-center cohort study involving low-risk PTMC patients treated with MWA in the Affiliated Hospital of Integrated Traditional Chinese and Western Medicine, Nanjing University of Chinese Medicine from August 2016 to October 2022 who were followed up for a minimum of 3 months (Fig. 1). Inclusion criteria were as follows: (i) PTMC with a single focus was diagnosed by thyroid fine-needle aspiration cytology (FNAC) or pathology by core needle biopsy; (ii) maximum tumor diameter ≤ 10 mm; (iii) aged 16–75 years; (iv) absence of clinical or imaging evidences of capsular invasion, extrathyroidal invasion, invasion of recurrent laryngeal nerve and trachea, lymph node metastasis, and distant metastasis; (v) subjects were clearly informed of the risk and willing to be treated with MWA. Exclusion criteria were as follows: (i) other types of thyroid cancers; (ii) PTMC with multiple foci; (iii) allergy to local anesthetics, painkillers, and hemostatics used in the present study; (iv) coagulation disorders (prothrombin time > 18 s, prothrombin activity < 40%); (v) pregnant and lactating women; (vi) clinical data were not available; (vii) combined with other malignant diseases. The study protocol was approved by the Ethics Committee of Affiliated Hospital of Integrated Traditional Chinese and Western Medicine, Nanjing University of Chinese Medicine. Written informed consent was obtained from all subjects.

### Definition

VRR was calculated using the formula: VRR (%) = (initial tumor volume − final volume)/initial volume × 100%. Two indexes, ablated volume (Va) and IAR, were introduced in the present study (22). Va was defined as the ablation area measured by contrast-enhanced ultrasonography on the day or the next day of MWA, which was calculated as follows: Va (cm^3^) = πabc/6. IAR was defined as the ratio of Va to preoperative tumor volume, which was calculated as follows: IAR (%) = Va/preoperative tumor volume × 100%.

The primary outcomes included local tumor progression, recurrence-free survival (RFS), and delayed surgery. Local tumor progression was defined as any of the following (23, 24): (i) malignancies persistently detected within the area where tumor ablation was confirmed by biopsy; (ii) newly formed PTMC lesions in the thyroid parenchyma confirmed by biopsy; (iii) cervical lymph node metastasis confirmed by ultrasound-guided puncture or surgery. RFS was defined as the duration from the primary treatment to the time of tumor relapse or the last follow-up visit. Delayed surgery was defined as when the patient having been initially treated with MWA received thyroidectomy due to tumor progression during the follow-up period or anxiety. The disappearance of tumor was defined as the absence of tumor in the ablation area confirmed by the follow-up ultrasonography.

Major and minor complications were recorded. The former mainly included recurrent laryngeal nerve injury and hypoparathyroidism, and the latter mainly included postoperative pain, bleeding, infection, and abnormal thyroid function. Recurrent laryngeal nerve injury was defined as the impairment of unilateral or bilateral vocal cord movement examined by laryngoscopy. Hypoparathyroidism was diagnosed by a parathyroid hormone level < 15 pg/mL at 24 h postoperatively. Permanent recurrent laryngeal nerve injury and hypoparathyroidism were defined as no recovery of the impairment of unilateral or bilateral vocal cord movement and parathyroid hormone level, respectively, within 6 months.

### MWA procedures

#### Preparations before MWA

Multi-angle cross-sectional ultrasound data of thyroid nodules were recorded using the Siemens ACUSON S2000 ultrasound system before MWA by experienced sonographers, including tumor size, volume, location, and surrounding tissues. These data were considered to determine the optimal approach to MWA.

#### Microwave ablation

Ultrasound-guided MWA was performed by an experienced physician using an MWA system (KY-2000, Canyon Medical, Nanjing, China) consisting of a generator, a power distribution system, and an antenna as previously reported (21). A cooled shaft antenna with 1.6-mm diameter, 10-cm length, and 3-mm distance from the electrode to the needle tip was used to puncture into the thyroid nodule. The MWA system produced 2450 MHz and 35 W output power to induce coagulative necrosis of thyroid tumors and the surrounding tissues, which were continuously ablated until the entire tumor and the surrounding area were hyperechoic. The residual heat of the antenna was used to coagulate the puncture approach to prevent the proliferation and implantation of tumor cells. We intermittently communicated with the patient during the MWA procedure, aiming to reduce the potential damage to the recurrent laryngeal nerve.

#### Follow-up

Thyroid function tests and ultrasound elastography were performed at 1, 3, 6, and 12 months, then every 6 months. Thyroid hormone suppression therapy was not given to PTMC patients after MWA. Recurrence, cervical lymph node metastasis, and distant metastasis were assessed, and Va, IAR, and VRR were calculated.

### Statistical analysis

Statistical analysis was performed using SPSS 20.0 and Prism. Measurement data in a normal distribution were expressed as mean ± standard deviation, otherwise as the median and quartile. Categorical data were expressed as the percentage. Differences between groups were analyzed by the Chi-square test or Fisher’s exact test. The cut-off value was identified by plotting ROC curves. The Kaplan–Meier curve was plotted to analyze the survival, followed by the comparison using the log-rank test. Univariable logistic regression analysis was performed to screen factors that were correlated with the recurrence risk of PTMC, and those with *P* < 0.2 were submitted to the multivariable logistic regression analysis to identify risk factors. *P* < 0.05 was considered statistically significant.

## Results

### Baseline characteristics

From August 2016 to October 2022, a total of 186 patients with PTMC were treated with MWA in our hospital. After the exclusion of 32 patients according to the inclusion criteria, 154 patients with PTMC were finally included in this study (Fig. 1). Patients were followed up for a median of 6 (3, 18) months, with a mean age of 38.43 ± 10.72 years, 92.21% younger than 55 years, and 78.57% being women. Finally, 49, 36, 27, 13, 15, 8, 3, and 3 patients were followed up for 3, 6, 12, 18, 24, 36, 48, and 60 months, respectively. The mean duration of MWA procedures was 100 (69, 126.5) s. The mean volume of thyroid nodules before MWA was 0.071 (0.039, 0.121) cm^3^, with a maximum tumor diameter of 0.60 ± 0.18 cm. The mean preoperative TSH level, postoperative Va, and postoperative IAR were 2.01 (1.28, 3.02) mU/L, 1.959 (1.144, 2.889) cm^3^ and 27.06 (13.54, 48.83), respectively (Table 1).

**Table 1:** Demographic characteristics and clinical parameters of the subjects.

Parameters	Results
Sex	
Female, *n* (%)	121 (78.57)
Male, *n* (%)	33 (21.43)
Age	
Age (years)	38.43 ± 10.72
<55, *n* (%)	142 (92.21)
≥55, *n* (%)	12 (7.79)
Location	
Left, *n* (%)	70 (45.45)
Right, *n* (%)	84 (54.55)
Ablation time (s)	100 (69, 126.5)
Maximum diameter (cm)	0.60 ± 0.18
Volume (cm^3^)	0.071 (0.039, 0.121)
Follow-up time (months)	6 (3, 18)
Pre-TSH	2.01 (1.28, 3.02)
Va (cm^3^)	1.959 (1.144, 2.889)
IAR	27.06 (13.54, 48.83)

IAR, initial ablation ratio; TSH, thyroid-stimulating hormone; Va, ablation area volume.

### Changes in tumor volume and VRR

**Figure 2:**
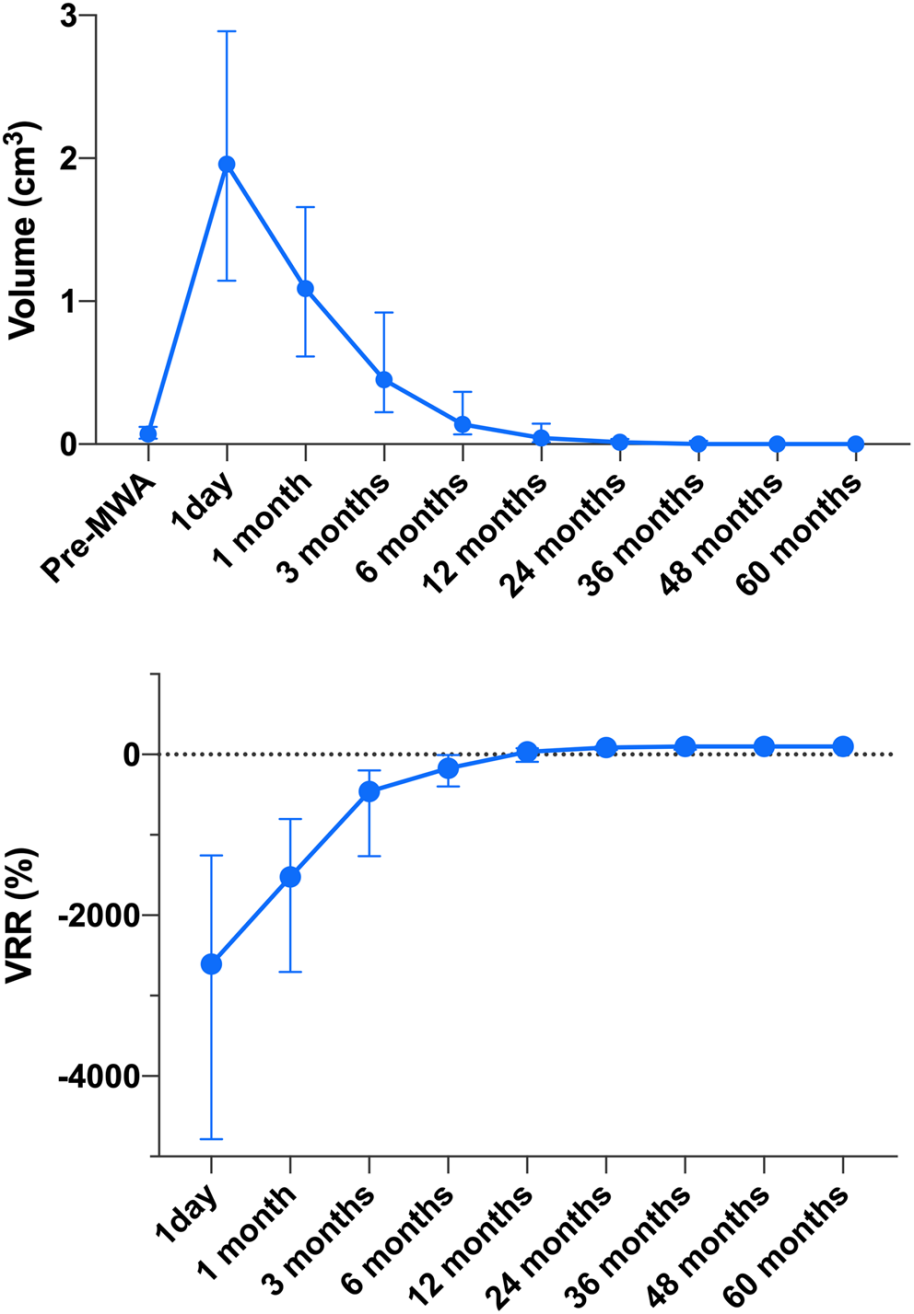
Changes in tumor volume and VRR from the baseline to 1, 3, 6, 12, 24, 36, 48, and 60 months after MWA. MWA, microwave ablation; VRR, volume reduction rate.

The median tumor volume of low-risk PTMCs before MWA was 0.071 mL, then 1.959, 1.089, 0.452, 0.140, 0.043, 0.000, and 0.000 mL at 1 day, 1 month, 3 months, 6 months, 12 months, 24 months, and 36 months after MWA, respectively (Table 2). Tumor ablation area increased immediately after MWA and then gradually shrunk. Tumor volume of PTMCs at 12 months postoperatively was significantly lower than that before MWA (*P* < 0.05). The median VRR at 24 months achieved 100%, which was maintained during a 60-month follow-up (Fig. 2).

**Table 2:** Changes in tumor volume and VRR over periods from the baseline to 1, 3, 6, 12, 24, 36, 48, and 60 months after MWA.

Time	Volume (cm^3^)	*P-*value	VRR (%)
Before MWA	0.071 (0.039, 0.122)		
1 day	1.959 (1.144, 2.889)	0.000	−2606.488 (−4783.013, −1254.477)
1 month	1.089 (0.613, 1.659)	0.000	−1504.275 (−2636.216, −788.475)
3 months	0.452 (0.223, 0.922)	0.000	−461.983 (−1266.005, −197.414)
6 months	0.140 (0.069, 0.365)	0.000	−169.284 (−396.124, −6.143)
12 months	0.043 (0.013, 0.144)	0.025	32.462 (−91.520, 78.266)
24 months	0.000 (0.000, 0.036)	0.000	100.000 (55.311, 100.000)
36 months	0.000 (0.000, 0.023)	0.000	100.000 (59.119, 100.000)
48 months	0.000 (0.000, 0.000)	0.001	100.000 (100.000, 100.000)
60 months	0.000 (0.000, 0.000)	0.003	100.000 (100.000, 100.000)

MWA, microwave ablation; VRR, volume reduction rate.

**Figure 3:**
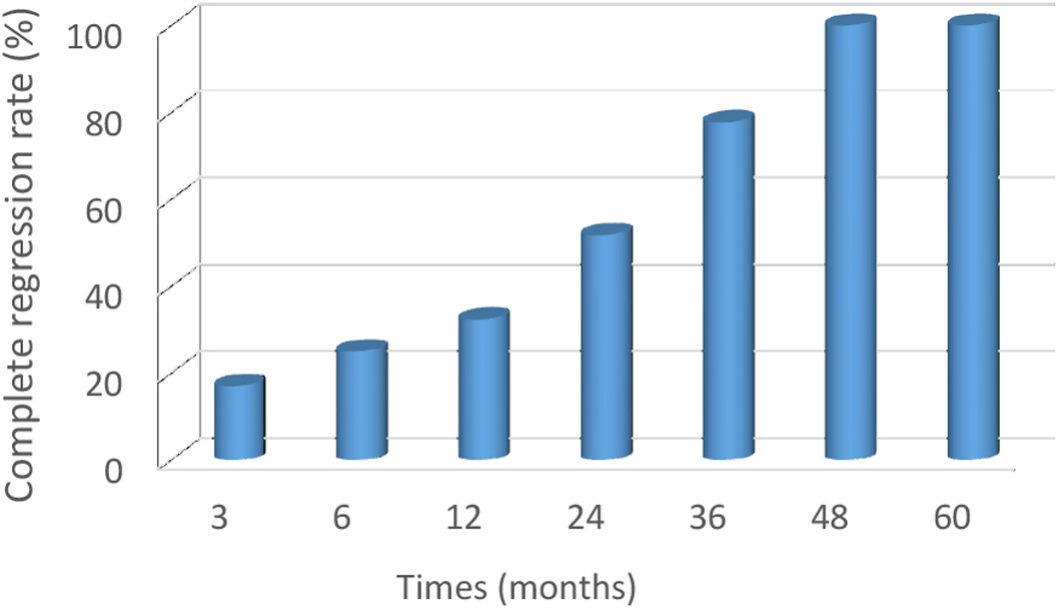
The rate of complete disappearance of tumor at each follow-up visit.

A complete disappearance of PTMC was observed in 25 patients during the follow-up period. The proportions of complete disappearance of tumor at 3, 6, 12, 24, 36, 48, and 60 months postoperatively were 17.01% (25/147), 25.00% (25/100), 32.31% (21/65), 51.72% (15/29), 77.78% (7/9), 100% (4/4), and 100% (3/3), respectively, showing a trend gradually increasing with the prolongation of the follow-up period (Fig. 3).

### Local tumor progression after MWA and risk factors

During the follow-up period, there were 7/154 (4.55%) cases of local tumor progression. Six patients received delayed surgery, and one was managed by AS. Suspicious nodule and/or lymph node metastasis were discovered by ultrasonography at 1 year of follow-up in Patients 1, 2, and 5, who were treated with salvage surgery. Patient 3 was treated with thyroidectomy due to the suspicious malignant findings obtained by the FNAC of the hypoechoic nodule primarily located in the thyroid isthmus at 1 year following MWA, which was pathologically diagnosed as PTMC with ipsilateral central lymph node metastasis. Abnormal findings were not detected on ultrasound images of Patient 4. However, Patient 4 was surgically treated because of anxiety. Tumor residue was not found in the ablation area, while lymph node metastasis was detected. Lymph node metastasis was detected in Patient 6 at 8 months, who was closely followed up. Patient 7 received salvage surgery due to the suspicious malignant findings obtained by the FNAC of the hypoechoic nodule located in the thyroid isthmus at 7 months. Generally, there were two cases of local recurrence, two cases of lymph node metastasis, and three cases of local recurrence combined with lymph node metastasis after MWA. Distant metastasis was not detected (Table 3).

**Table 3:** Characteristics of seven PTMC patients with local tumor progression.

No.	Sex	Age	Location	RFS	Treatment	Type of local tumor progression
1	Female	40	Left	12	TT	New PTMC
2	Female	29	Left	12	TT	New PTMC + LNM
3	Male	29	Right	3	LT	Residual tumor + LNM
4	Female	33	Left	12	LT	LNM
5	Male	47	Left	12	LT	New PTMC + LNM
6	Female	20	Right	8	AS	LNM
7	Female	45	Left	7	TT	New PTMC

AS, active surveillance; LNM, lymph node metastasis; LT, thyroid lobectomy; MWA, microwave ablation; PTMC, papillary thyroid microcarcinoma; RFS, recurrence-free survival; TT, total thyroidectomy.

**Figure 4:**
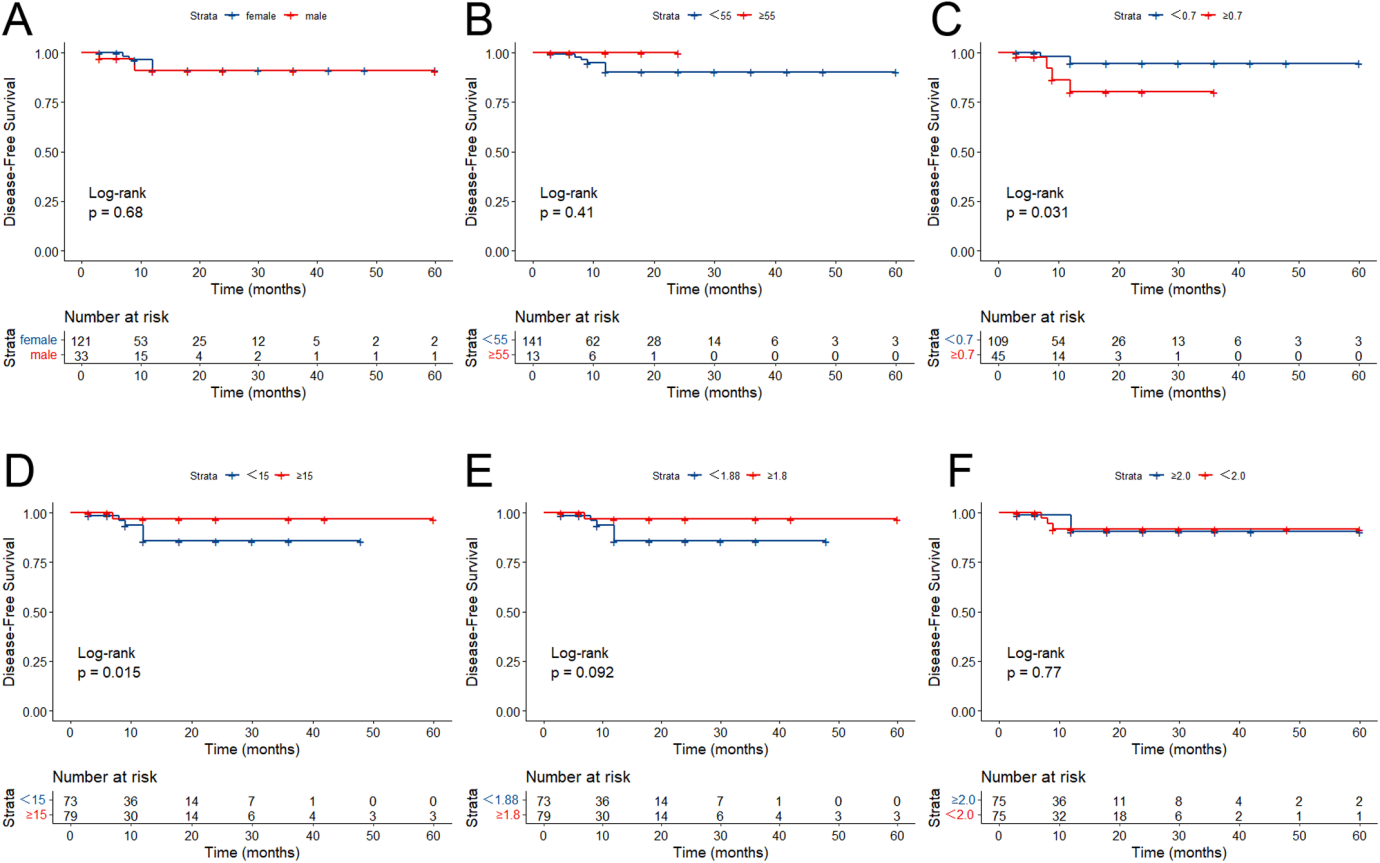
Recurrence-free survival curves in MWA-treated low-risk PTMC patients subgrouped by sex (A), age (B), maximum tumor diameter (C), initial ablation ratio (D), ablated volume (E), and preoperative TSH (F). MWA, microwave ablation; PTMC, papillary thyroid microwave ablation; TSH, thyroid-stimulating hormone.

We further performed Kaplan–Meier survival analysis based on the common risk factors for local tumor progression, including sex, age, and maximum tumor diameter (Fig. 4). Subgrouped by sex, RFS was comparable between male and female PTMC patients (*P* = 0.68). None of the PTMC patients older than 55 years suffered from tumor recurrence, although no significant difference in RFS was detected between PTMC patients younger and older than 55 years (*P* = 0.41). Based on the cut-off value of 0.70 cm obtained by the plotted ROC curves, a significantly lower RFS was detected in PTMC patients with a maximum tumor diameter < 0.70 cm than those with ≥0.70 cm (*P* = 0.031). However, the area under the curve was relatively small (AUC = 0.565), suggesting the poor potential of the maximum tumor diameter in diagnosing PTMC. RFS was comparable between PTMC patients subgrouped by Va with a cut-off value of 1.88 cm^3^ (*P* = 0.092). PTMC patients with IAR ≥ 15 had a better prognosis than those with IAR < 15 (*P* = 0.015, AUC = 0.672). After excluding PTMC patients with a history of hyperthyroidism or hypothyroidism (*n* = 4) and those lacking follow-up data (*n* = 4), the remaining were classified into two groups based on the preoperative TSH level of 2.0 mU/L. We did not detect a significant difference in RFS between PTMC patients with TSH ≤ 2.0 mU/L and >2.0 mU/L (*P* = 0.77).

### Influencing factors for tumor disappearance after MWA

Clinical data of PTMC patients were introduced into the univariable logistic regression model, and the follow-up time, tumor volume, maximum tumor diameter, ablation time, free thyroxine, and IAR were found to be significantly correlated with the disappearance of PTMC after MWA. These factors were further entered into the multivariable logistic regression model. It is shown that the follow-up time was an independent protective factor for the disappearance of PTMC after MWA (*P* = 0.044; OR, 0.940; 95% CI (0.886, 0.998)), while IAR was a risk factor (*P* = 0.036; OR, 1.029; 95% CI (1.002, 1.057)) (Table 4).

**Table 4:** Logistic regression analysis of factors affecting the disappearance of PTMC.

Factors	Univariate analysis			Multivariate analysis		
	B	OR (95% CI)	*P*	B	OR (95% CI)	*P*
Follow-up time	−0.081	0.922 (0.880, 0.967)	0.001	−0.062	0.940 (0.886, 0.998)	0.044
Volume	7.166	1294.487 (0.544, 3,081,979.241)	0.071	11.689	119,225.117 (0.000, 4.099 × 10^15^)	0.345
Maximum diameter	2.437	11.441 (0.731, 179.025)	0.082	0.947	2.578 (0.001, 9228.064)	0.821
FT4	0.151	1.163 (1.018, 1.329)	0.027	0.174	1.19 (0.981, 1.443)	0.078
Ablation time	0.025	1.025 (1.009, 1.041)	0.002	0.012	1.012 (0.993, 1.030)	0.214
IAR	0.015	1.015 (0.997, 1.033)	0.101	0.029	1.029 (1.002, 1.057)	0.036

FT4, free thyroxine; IAR, initial ablation ratio; PTMC, papillary thyroid microcarcinoma.

### Complications

No patient experienced intolerable pain or significant discomfort which would have required treatment suspension during MWA procedures. Four patients suffered hoarseness during the follow-up period, with three having recovered within 1 week and one after 6 months. Preoperative abnormal thyroid function was detected in 19 PTMC patients who recovered after the corresponding treatment. The incidences of abnormal thyroid function at 3, 6, 12, and 24 months after MWA were 12.84% (14/109), 15.19% (12/79), 18% (9/50), and 4.5% (1/22), respectively.

## Discussion

Surgery or AS is the conventional treatment for low-risk PTMC, although the former may induce overtreatment and complications, and AS is only suitable for patients with certain indications. The application of AS is still controversial, especially in developing countries (25). The therapeutic efficacy of MWA on PTMC has been previously validated as a reliable alternative to surgery and AS. Nevertheless, it is difficult to predict local tumor progression.

Our results consistently confirmed that MWA was an effective treatment for low-risk PTMC. The tumor volume was significantly reduced at 12 months after MWA. At 24 months, the median VRR achieved 100%, which was maintained during a 60-month follow-up. A total of 154 patients were followed up for more than 3 months. The rate of local tumor progression was 4.55%, slightly higher than that previously reported in other centers (0–4.2%) (26, 27, 28). Kim *et al.* (19) have reported that the rate of local tumor progression after LA is 5.5%, and local tumor progression occurs in most cases within 5 years. Therefore, we recommend a close follow-up of 5 years for MWA. In the present study, suspicious tumor lesions or lymph node metastases were detected by the follow-up ultrasonography within 1 year of MWA in five PTMC patients with local tumor progression, all of which were further confirmed by FNA or postoperative pathological examination. As a result, a close follow-up by cervical ultrasonography within 1 year of MWA is also recommended.

Sex, age, and tumor size are considered as risk factors for lymph node metastasis in PTMC patients (29, 30, 31). Our results showed that the RFS was comparable between male and female PTMC patients, although most of the recruited patients were women. Age < 55 years is believed as an independent risk factor for lymph node metastasis in PTMC (30). Ito *et al.* (15) have found that during AS in PTMC patients, older patients have a lower risk of tumor progression, especially those older than 60 years. According to the AJCC/UICC 8th edition, the cut-off value of 55 years was adopted. It is shown that PTMC patients ≥ 55 years had a better prognosis, although no significant difference was detected in the RFS between those younger and older than 55 years. It is suggested that the risk of local tumor progression was relatively low in elderly patients with PTMC during the treatment of MWA and AS. FNA is not recommended for patients with a maximum tumor diameter < 0.5 cm by the American Thyroid Association (ATA) (32). Therefore, the maximum tumor diameter of almost all recruited PTMC patients in the present study was larger than 0.5 cm. Subgrouped by the cut-off value of 0.7 cm, PTMC patients with a maximum tumor diameter < 0.7 cm presented a better prognosis. Sim *et al.* (22) have calculated the IAR to quantitatively assess the therapeutic efficacy of thermal ablation on benign thyroid nodules, finding that IAR is highly correlated with VRR. As a larger ablation area is more likely to completely inactivate the tumor lesion, we introduced Va and IAR to evaluate the RFS. It is shown that PTMC patients with IAR ≥ 15 had a better prognosis. Therefore, a larger ablation area was favorable to reduce the postoperative recurrence risk of PTMC. Thyroid hormone suppression therapy after thyroid lobectomy significantly improves the prognosis of high-risk differentiated thyroid cancer and prevents its progression or recurrence. Whether low-risk patients can benefit from it, however, remains unclear (33). The ATA guidelines recommend that the TSH level in low-risk PTC patients treated with thyroid lobectomy maintains in the middle or low reference range (0.5–2mU/L) but maintains in a low reference range in patients managed by AS. Li *et al.* (23) have reported that there is no significant difference in the local tumor progression between low-risk PTC patients who have high and low TSH levels after RFA (9.4 vs 4.3%). Consistently, our data revealed that the RFS was similar between PTMC patients with preoperative TSH ≤ 2.0 mU/L and >2.0 mU/L. Recent studies have shown that the postoperative TSH level is not correlated with the recurrence in low-risk PTC patients after thyroid lobectomy (34, 35). Therefore, thyroid hormone suppression therapy, or even keeping TSH ≤ 2.0 mU/L, is not necessary for low-risk PTMC patients after MWA, as long as normal thyroid function is maintained.

During the follow-up period, the rate of complete disappearance of tumor was 17.01% (25/147), which was lower than the previously reported 57.6–95.2% (36, 37). It may be attributed to the definition of tumor disappearance in the present study as the complete disappearance of the ablated area confirmed by ultrasonography, excluding the scar-like changes (38). A relatively short follow-up period during which the ablation area had not disappeared could be another cause. Logistic regression analysis showed that the follow-up time was a protective factor for the complete disappearance of tumor, which is consistent with the changing rate of tumor disappearance. IAR was identified as a risk factor for the disappearance of the ablation area, which may be attributed to the coagulation necrosis of the tumor due to MWA-induced vibration of water molecules, and denatured proteins caused by thermal deposition of surrounding tissues that were not easily absorbed (39).

MWA may cause subcutaneous neck hematoma, fever, pain, voice change, skin burns, edema, and hypothyroidism. Severe complications of MWA include permanent recurrent laryngeal nerve damage and hypoparathyroidism. Through the literature review, the complication rate of PTMC treated with MWA ranges from 0 to 5.2% (18, 28). In our study, the incidence of hoarseness after MWA was 2.60% (4/154), and hypoparathyroidism was not reported. The incidence of abnormal thyroid function after MWA was significantly high, which may be attributed to the exclusion of patients with lymph node metastasis by preoperative enhanced CT. The use of iodinated contrast media would increase the risk of thyroid dysfunction (40).

Some limitations in the present study should be noted. First of all, it was a single-center retrospective study. Second, the follow-up time of most PTMC patients was not long enough to effectively evaluate the long-term efficacy and safety of MWA. Third, most of the recruited PTMC patients were women and younger than 55 years, which may cause potential biases. Fourth, the number of local tumor progression was small, which may influence the statistical power of the analysis of its risk factors. Therefore, our findings should be further validated in multi-center studies with a large sample size and a long follow-up period.

Collectively, our study validated that ultrasound-guided MWA is a safe and effective therapeutic strategy for low-risk PTMC. The maximum tumor diameter before MWA and IAR are predictors of local tumor progression, and the follow-up time and IAR are significantly correlated with the complete disappearance of the tumor in the ablation area.

## Declaration of interest

The authors declare that they have no competing interest.

## Funding

This study was funded by the Jiangsu Provincial Key Research and Development Programhttp://dx.doi.org/10.13039/501100013058 (BE2020726), the Medical Scientific Research Foundation of Jiangsu Province of China (M2020102), and Young & Middle-aged Doctors of China International Medical Exchange Foundation (BQE-JZX-202115).

## Availability of data and material

All data are made available by the authors upon request without undue reservation.

## Author contribution statement

Yujie Ren, Lin Jiang, and Shuhang Xu developed the research questionnaire and drafted the protocol for this study. Yujie Ren, Xue Han, Yujiang Li, Shuhang Xu, and Chao Liu were responsible for data collection and analysis. Guofang Chen and Shuhang Xu participated in the diagnosis. Shuhang Xu performed microwave ablation. Xue Han was responsible for the perioperative management. Yujie Ren and Yujiang Li drafted the manuscript. Lin Jiang and Shuhang revised the manuscript critically for important intellectual content. All authors agreed to take responsibility for the integrity of the data and the accuracy of data analysis, and approved the final version of the manuscript.

## References

[1] Li M Dal Maso L & Vaccarella S. Global trends in thyroid cancer incidence and the impact of overdiagnosis. Lancet. Diabetes and Endocrinology 2020 8 468–470. (10.1016/S2213-8587(2030115-7) 32445733

[2] Sung H Ferlay J Siegel RL Laversanne M Soerjomataram I Jemal A & Bray F. Global cancer statistics 2020: GLOBOCAN estimates of incidence and mortality worldwide for 36 cancers in 185 countries. CA 2021 71 209–249. (10.3322/caac.21660) 33538338

[3] Megwalu UC & Moon PK. Thyroid cancer incidence and mortality trends in the United States: 2000–2018. Thyroid 2022 32 560–570. (10.1089/thy.2021.0662) 35132899

[4] Du L Wang Y Sun X Li H Geng X Ge M & Zhu Y. Thyroid cancer: trends in incidence, mortality and clinical-pathological patterns in Zhejiang Province, Southeast China. BMC Cancer 2018 18 291. (10.1186/s12885-018-4081-7) 29544469 PMC5856225

[5] Xie L Wang S Qian Y Jia S Wang J Li L Zhang W Yu H Bao P & Qian B. Increasing gap between thyroid cancer incidence and mortality in urban Shanghai, China: an analysis spanning 43 years. Endocrine Practice 2021 27 1100–1107. (10.1016/j.eprac.2021.06.002) 34119680

[6] Pizzato M Li M Vignat J Laversanne M Singh D La Vecchia C & Vaccarella S. The epidemiological landscape of thyroid cancer worldwide: GLOBOCAN estimates for incidence and mortality rates in 2020. Lancet. Diabetes and Endocrinology 2022 10 264–272. (10.1016/S2213-8587(2200035-3) 35271818

[7] Yu XM Wan Y Sippel RS & Chen H. Should all papillary thyroid microcarcinomas be aggressively treated? An analysis of 18,445 cases. Annals of Surgery 2011 254 653–660. (10.1097/SLA.0b013e318230036d) 21876434

[8] Hsiao V Light TJ Adil AA Tao M Chiu AS Hitchcock M Arroyo N Fernandes-Taylor S & Francis DO. Complication rates of total thyroidectomy vs hemithyroidectomy for treatment of papillary thyroid microcarcinoma: a systematic review and meta-analysis. JAMA Otolaryngology– Head and Neck Surgery 2022 148 531–539. (10.1001/jamaoto.2022.0621) 35511129 PMC9073663

[9] Mehanna H Al-Maqbili T Carter B Martin E Campain N Watkinson J McCabe C Boelaert K & Franklyn JA. Differences in the recurrence and mortality outcomes rates of incidental and nonincidental papillary thyroid microcarcinoma: a systematic review and meta-analysis of 21 329 person-years of follow-up. Journal of Clinical Endocrinology and Metabolism 2014 99 2834–2843. (10.1210/jc.2013-2118) 24828487

[10] Wen X Jin Q Cen X Qiu M & Wu Z. Clinicopathologic predictors of central lymph node metastases in clinical node-negative papillary thyroid microcarcinoma: a systematic review and meta-analysis. World Journal of Surgical Oncology 2022 20 106. (10.1186/s12957-022-02573-7) 35365171 PMC8976349

[11] Sugitani I Ito Y Takeuchi D Nakayama H Masaki C Shindo H Teshima M Horiguchi K Yoshida Y Kanai T, Indications and strategy for active surveillance of adult low-risk papillary thyroid microcarcinoma: consensus statements from the Japan association of endocrine surgery task force on management for papillary thyroid microcarcinoma. Thyroid 2021 31 183–192. (10.1089/thy.2020.0330) 33023426 PMC7891203

[12] Tuttle RM Fagin JA Minkowitz G Wong RJ Roman B Patel S Untch B Ganly I Shaha AR Shah JP, Natural history and tumor volume kinetics of papillary thyroid cancers during active surveillance. JAMA Otolaryngology– Head and Neck Surgery 2017 143 1015–1020. (10.1001/jamaoto.2017.1442) 28859191 PMC5710258

[13] Oh HS Ha J Kim HI Kim TH Kim WG Lim DJ Kim TY Kim SW Kim WB Shong YK, Active surveillance of low-risk papillary thyroid microcarcinoma: a multi-center cohort study in Korea. Thyroid 2018 28 1587–1594. (10.1089/thy.2018.0263) 30226447

[14] Ito Y Uruno T Nakano K Takamura Y Miya A Kobayashi K Yokozawa T Matsuzuka F Kuma S Kuma K, An observation trial without surgical treatment in patients with papillary microcarcinoma of the thyroid. Thyroid 2003 13 381–387. (10.1089/105072503321669875) 12804106

[15] Ito Y Miyauchi A Kihara M Higashiyama T Kobayashi K & Miya A. Patient age is significantly related to the progression of papillary microcarcinoma of the thyroid under observation. Thyroid 2014 24 27–34. (10.1089/thy.2013.0367) 24001104 PMC3887422

[16] Ito Y Miyauchi A & Oda H. Low-risk papillary microcarcinoma of the thyroid: a review of active surveillance trials. European Journal of Surgical Oncology 2018 44 307–315. (10.1016/j.ejso.2017.03.004) 28343733

[17] Oh HS Park S Kim M Kwon H Song E Sung TY Lee YM Kim WG Kim TY Shong YK, Young age and male sex are predictors of large-volume central neck lymph node metastasis in clinical N0 papillary thyroid microcarcinomas. Thyroid 2017 27 1285–1290. (10.1089/thy.2017.0250) 28741452

[18] Yan L Zhang M Song Q & Luo Y. Ultrasound-guided radiofrequency ablation versus thyroid lobectomy for low-risk papillary thyroid microcarcinoma: a propensity-matched cohort study of 884 patients. Thyroid 2021 31 1662–1672. (10.1089/thy.2021.0100) 34269611

[19] Kim HJ Chung SM Kim H Jang JY Yang JH Moon JS Son G Oh JR Bae JY & Yoon H. Long-term efficacy of ultrasound-guided laser ablation for papillary thyroid microcarcinoma: results of a 10-year retrospective study. Thyroid 2021 31 1723–1729. (10.1089/thy.2021.0151) 34445885

[20] Wei Y Niu WQ Zhao ZL Wu J Peng LL Li Y & Yu MA. Microwave ablation versus Surgical Resection for Solitary T1N0M0 Papillary thyroid Carcinoma. Radiology 2022 304 704–713. (10.1148/radiol.212313) 35536133

[21] Lu C Li X Chu X Li R Li J Wang J Wang Y Xu Y Chen G Xu S, Clinical effects of microwave ablation in the treatment of low-risk papillary thyroid microcarcinomas and related histopathological changes. Frontiers in Endocrinology (Lausanne) 2021 12 751213. (10.3389/fendo.2021.751213) PMC8482998 34603216

[22] Sim JS Baek JH & Cho W. Initial ablation ratio: quantitative value predicting the therapeutic success of thyroid radiofrequency ablation. Thyroid 2018 28 1443–1449. (10.1089/thy.2018.0180) 30226441

[23] Li X Yan L Xiao J Li Y Zhu Y Yang Z Zhang M & Luo Y. Optimal thyrotropin level for low-risk papillary thyroid carcinoma after ultrasound-guided radiofrequency ablation. International Journal of Hyperthermia 2023 40 2160880. (10.1080/02656736.2022.2160880) 36599433

[24] Zhang M Tufano RP Russell JO Zhang Y Zhang Y Qiao Z & Luo Y. Ultrasound-guided radiofrequency ablation versus surgery for low-risk papillary thyroid microcarcinoma: results of over 5 years' follow-up. Thyroid 2020 30 408–417. (10.1089/thy.2019.0147) 31910107

[25] Cernea CR Matos LL Eugenio C Ferreira GM Cerqueira YS Leite AKN Vanderlei FAB de Carlucci D Gotoda RN Hojaij FC, Active surveillance of thyroid microcarcinomas: a critical view. Current Oncology Reports 2022 24 69–76. (10.1007/s11912-021-01177-w) 35061193

[26] Teng DK Li WH Du JR Wang H Yang DY & Wu XL. Effects of microwave ablation on papillary thyroid microcarcinoma: a five-year follow-up report. Thyroid 2020 30 1752–1758. (10.1089/thy.2020.0049) 32458748

[27] Yue WW Qi L Wang DD Yu SJ Wang XJ Xu HX & Wang SR. US-guided microwave ablation of low-risk papillary thyroid microcarcinoma: longer-term results of a prospective study. Journal of Clinical Endocrinology and Metabolism 2020 105. (10.1210/clinem/dgaa128) 32198508

[28] Li J Liu Y Liu J Yang P Hu X & Qian L. A comparative study of short-term efficacy and safety for thyroid micropapillary carcinoma patients after microwave ablation or surgery. International Journal of Hyperthermia 2019 36 640–646. (10.1080/02656736.2019.1626492) 31244350

[29] Feng JW Ye J Wu WX Qu Z Qin AC & Jiang Y. Management of cN0 papillary thyroid microcarcinoma patients according to risk-scoring model for central lymph node metastasis and predictors of recurrence. Journal of Endocrinological Investigation 2020 43 1807–1817. (10.1007/s40618-020-01326-1) 32557354

[30] Yin Y Xu X Shen L Zhao W Diao H & Li C. Influencing factors and cumulative risk analysis of cervical lymph node metastasis of papillary thyroid microcarcinoma. Frontiers in Oncology 2021 11 644645. (10.3389/fonc.2021.644645) 34660255 PMC8514816

[31] Ye J Feng JW Wu WX Hu J Hong LZ Qin AC Shi WH & Jiang Y. Papillary thyroid microcarcinoma: a nomogram based on clinical and ultrasound features to improve the prediction of lymph node metastases in the central compartment. Frontiers in Endocrinology (Lausanne) 2021 12 770824. (10.3389/fendo.2021.770824) PMC8790095 35095755

[32] Haugen BR Alexander EK Bible KC Doherty GM Mandel SJ Nikiforov YE Pacini F Randolph GW Sawka AM Schlumberger M, 2015 American Thyroid Association management guidelines for adult patients with thyroid nodules and differentiated thyroid cancer: the American Thyroid Association guidelines task force on thyroid nodules and differentiated thyroid cancer. Thyroid 2016 26 1–133. (10.1089/thy.2015.0020) 26462967 PMC4739132

[33] Won HR Jeon E Chang JW Kang YE Song K Kim SW Lim DM Ha TK Chung KW Kim HJ, Is maintaining thyroid-stimulating hormone effective in patients undergoing thyroid lobectomy for low-risk differentiated thyroid cancer? A systematic review and meta-analysis. Cancers (Basel) 2022 14. (10.3390/cancers14061470) PMC8946503 35326621

[34] Xu S Huang Y Huang H Zhang X Qian J Wang X Xu Z Liu S & Liu J. Optimal serum thyrotropin level for patients with papillary thyroid carcinoma after lobectomy. Thyroid 2022 32 138–144. (10.1089/thy.2021.0404) 34617446

[35] Lee MC Kim MJ Choi HS Cho SW Lee GH Park YJ & Park DJ. Postoperative thyroid-stimulating hormone levels did not affect recurrence after thyroid lobectomy in patients with papillary thyroid cancer. Endocrinology and Metabolism 2019 34 150–157. (10.3803/EnM.2019.34.2.150) 31099202 PMC6599911

[36] Choi Y & Jung SL. Efficacy and safety of thermal ablation techniques for the treatment of primary papillary thyroid microcarcinoma: a systematic review and meta-analysis. Thyroid 2020 30 720–731. (10.1089/thy.2019.0707) 31801432

[37] Ou D Chen C Jiang T & Xu D. Research review of thermal ablation in the treatment of papillary thyroid carcinoma. Frontiers in Oncology 2022 12 859396. (10.3389/fonc.2022.859396) 35847945 PMC9283792

[38] Cho SJ Baek JH Chung SR Choi YJ & Lee JH. Thermal ablation for small papillary thyroid cancer: a systematic review. Thyroid 2019 29 1774–1783. (10.1089/thy.2019.0377) 31739738

[39] Yin L Li XY Zhu LL Chen GL Xiang Z Wang QQ Bi JW & Wang Q. Clinical application status and prospect of the combined anti-tumor strategy of ablation and immunotherapy. Frontiers in Immunology 2022 13 965120. (10.3389/fimmu.2022.965120) 36131929 PMC9483102

[40] Kornelius E Chiou JY Yang YS Peng CH Lai YR & Huang CN. Iodinated contrast media increased the risk of thyroid dysfunction: a 6-year retrospective cohort study. Journal of Clinical Endocrinology and Metabolism 2015 100 3372–3379. (10.1210/JC.2015-2329) 26168278

